# Pharmacists' Research Contributions in the Fight against HIV/AIDs

**DOI:** 10.1155/2012/869891

**Published:** 2012-10-31

**Authors:** Alexis E. Horace

**Affiliations:** College of Pharmacy, University of Louisiana at Monroe, Baton Rouge Campus, 3849 North Boulevard, Baton Rouge, LA 70808, USA

## Abstract

Pharmacists have made many contributions to HIV/AIDs research and are still showing their significance as members of the healthcare team through innovative clinical trials. Pharmacists are showing advances in several healthcare settings including inpatient, outpatient, and community pharmacies. Because of the complex regimens of highly active antiretroviral therapy (HAART), the increased life span of patients living with HIV, and other concomitant medications taken for comorbid disease states, there is a high risk for health-related complications and the development of adverse events. These adverse events may lead to decreased adherence to HAART, which may cause the development of HIV drug resistance. Pharmacists are providing examples through growing research on how they help combat medication-related errors and also continue to contribute as healthcare providers as a part of a holistic healthcare team.

## 1. Introduction

With the invention and administration of highly active antiretroviral therapy (HAART), human immunodeficiency virus (HIV) has transformed from an acute disease to a chronic disease. The Centers for Disease Control and Prevention estimate that between the years 1996–2003, HAART extended life expectancy of patients living with HIV from 10 to 20 years [1]. Care for patients infected with HIV has shifted from primarily requiring acute treatment in the inpatient setting to needing chronic treatment in the primary and ambulatory care settings. During this time, pharmacists have assumed larger roles as members of healthcare teams who specialize in caring for patients of this population. The responsibilities of pharmacists surpass caring for HIV patients in a community pharmacy setting and have evolved into participating in direct patient care in hospitals and outpatient clinics.

Concerns for patients with HIV extend beyond increasing CD4 cell counts and decreasing viral loads. Successful treatment for patients with this disease state depends on a holistic, patient-specific approach by a multidisciplinary team [9]. Management of all aspects of HAART is needed for successful treatment. Clinical pharmacists can provide many services such as pharmacokinetic drug monitoring, medication reconciliation, therapeutic medication recommendations, patient counseling, adherence consult services, and medication therapy management. These services have prevented medication errors, decreased medication misuse, and increased health-related outcomes [10–12]. Due to the complexity of HAART, there is a greater need to monitor adverse effects, drug-drug interactions, and resistance patterns of HIV-1 virus that can develop through patient nonadherence.

Patients may require treatment for chronic disease states such as hepatitis coinfection, or, solid organ transplant, diabetes, cardiovascular disease, or renal disease, which also use many medications for treatment further increasing the chance for drug interactions to occur.

Pharmacists have demonstrated their usefulness in the clinical setting through providing cost savings by making valuable therapeutic interventions [13–15]. Kopp and colleagues showed pharmacists' interventions over 4.5 months of service provided a cost avoidance of $205,919–$280,421 [16]. Not only do pharmacists play a vital role as a part of the healthcare team providing optimal care for patients with HIV, but also they are a cost-effective resource.

The role of pharmacists treating patients with HIV is constantly evolving. The American Society of Health System Pharmacists (ASHP) released a statement in 2003 that supports the use of clinical pharmacists in caring for patients with this disease state [17]. ASHP suggests that possible areas for implementation of pharmacists include outpatient pharmacies, ambulatory care clinics, inpatient settings, dialysis units, hospice care centers, and home health services. Since the publication of this statement, there has been a plethora of studies documenting the contributions of pharmacists in the fight against HIV. This paper reviews efforts of pharmacists and pharmacy services to aid in the management of this disease state and provides insight into areas for expanding future research.

## 2. Hospital Pharmacy

Patients with chronic disease states are at risk for medication-related errors upon admission to the hospital [18]. Drug interactions with HAART can significantly increase or decrease therapeutic levels of medications [19] putting patients at risk for adverse effects or the development of HIV-1 viral resistance. An adherence rate of 95% to antiretroviral (ARV) therapy is recommended in order to sustain acceptable CD4 counts and viral load levels [20]. The medication-related errors that occur during inpatient hospital stays are more detrimental than they may appear on the surface [20]. Early retrospective studies documenting the need for pharmacy intervention report clinically significant error rates in prescribing ARVs of 5.8% over a 2-year period and 26% over a 1-year period [21, 22]. Because of the impact these errors can make on the health of HIV positive patients, research has emerged showing how the participation of pharmacists decreases these risks.

In 2007, Heelon and colleagues [23] published data describing the AV prescribing errors in an inpatient hospital setting and the effects of a clinical pharmacist in decreasing these errors. This descriptive, observational study was constructed in two parts consisting of a preintervention phase and an intervention phase. They found that 21% of HIV patients had at least one HAART prescribing error during their hospital stay. Most errors consisted of incomplete regimens (45%), incorrect dosage forms (30%), and incorrect scheduling (8%). With the help of a pharmacist, the duration of errors also decreased from 3.5 days to 1 day until resolution.

That same year, another study examined the effect of clinical pharmacy on health outcomes for HIV patients [12]. This observational study reviewed 1571 patients in clinics with or without a clinical pharmacist's involvement. Patients with access to a clinical pharmacist (47%) were associated with decreases in plasma HIV-1 virus levels of 0.73 log (*P* < 0.001) within 1 year and 0.33 log (*P* = 0.005) within 2 years. There were no statistically significant changes in CD4 T-cell counts after 2 years. Outcomes varied depending on the size of the practice site. For instance, the clinical pharmacist group associated with patient population of <50 were associated with a 19% (95% CI: −40% to 8%) decrease in office visits; however, for sites with panel sizes >50 HIV patients, there was a 10% increase (95% CI: −16% to 43%). Patients who had access to a clinical pharmacist required fewer clinic visits (0.88 fewer, *P* < 0.001). In addition, adherence rates in patients in the clinical pharmacists group improved to 7.1% at 1 year (81% versus 74%, *P* = 0.04) and 7.8% at 2 years (76.7% versus 68.9%, *P* = 0.02).

In the years that followed, other research on the impact of clinical pharmacists in the hospital setting emerged. A small study published by Mok and colleagues supports previous data reporting high rates of incomplete regimens for HIV patients admitted to the hospital (36% of patients, *n* = 83) and 86% of patients had at least one medication error involving HAART [24]. Also, a 3-month descriptive, prospective study conveyed similar results reporting rates of incomplete regimens in hospitalized patients as high as 42% (*n* = 50) and, with the aid of clinical pharmacists' recommendations, all patients admitted were started on appropriate regimens (*n* = 34) [25]. Another study reflected similar data [26]. However, the authors rated the risk level of each error. In a population of 68 HIV patients hospitalized over a 4-month period, 56% had at least one error that caused moderate-to-severe discomfort. Another interesting aspect of this study was the authors ability to associate an approximately 2-fold increase in the risk of experiencing HIV medication errors with the hospital pharmacy's inability to provide correct substitutions for nonformulary medications (RR = 1.95; 95% CI 1.25 to 3.4; *P* = 0.02). Not only did this study provide insight on the severity of errors detected and the importance of a pharmacist's intervention on these errors, but also it provided information on improvements the department of pharmacy could implement when dispensing AVs.

A larger study emerged in 2011 by Carcelero and colleagues identifying common prescribing errors involving AVs, and also, evaluating the level of acceptance of pharmacy-provided recommendations [27]. This observational, prospective study was conducted over 1 year and included 189 HIV-infected patients. Similar rates of medication-related errors were found in comparison to previous studies (21.7% of patients had at least one error). The pharmacist made an intervention for all detected errors with 91.7% of recommendations being accepted. The data presented supports the need for the incorporation of clinical pharmacist in the care for patients hospitalized with HIV/AIDS. Services provided by clinical pharmacists should be taken into consideration in this practicing setting.

## 3. Outpatient Clinics and Community ****Pharmacies

Care for patients with HIV/AIDs extends beyond the inpatient setting to outpatient settings as rates for developing opportunistic infections continue to decrease [28]. There are now opportunities for pharmacists to participate in the continuity of care for these patients as they transition from one area of healthcare to another. One study reports discrepancy rates as high as 53% when comparing community pharmacy and outpatient clinic medication records for patients on AVs [29]. In addition to the monitoring patient tolerance of these complex ARV regimens and identifying drug-drug interactions between ARVs and medications taken for other chronic disease states, pharmacists also have opportunities to assist in adherence counseling and help to optimize drug therapy. Taking AVs is a long-term commitment and requires excellent adherence. The level of patient adherence is crucial in optimizing therapeutic outcomes [20]. Length of therapy, psychological comorbidities, larger pill burden, increased frequency of administration, and baseline viral loads with associated resistance patterns are determinants of adherence [30]. In a retrospective study (*n* = 80) that reviews adherence by use of refill data, more patients receiving counseling from a pharmacist refilled their prescriptions in a timely manner than those who did not (*P* < 0.05) [31].

March and colleagues published a study in 2007 that showed data on the effects of pharmacists' interventions on patient outcomes in an HIV clinic [32]. Patients recruited to this study had an extensive history with AV therapy. In this observational trial which lasted approximately 4 months, 68% of the patients (23 out of 34) had more than one medication problem that required therapeutic recommendations from the pharmacist. During this time, the clinic's primary care providers accepted 100% of recommendations. When observing CD4+ cells, they found counts increased from baseline levels by 54± cells/mm^3^ (*P* < 0.0002) over the course of the study. The mean reduction in viral load was 1.02 log_10_ copies/mL (*P* < 0.004). Though this study is small, it shows that the help of a clinical pharmacist has some benefits. This study also highlights an area for further research for pharmacists hoping to make further impacts on the fight against HIV.

There are several other studies published that provide additional support to the use of pharmacy when caring for patients with HIV (Table 1). Not only are clinical pharmacists vital in the clinical setting, but also the use of specialty pharmacies for HIV patients or pharmacist-run medication therapy management (MTM) clinics have made a great impact. Opportunities for improvement lie in continuing to document pharmacy-related services to show health outcomes and cost savings in relation to pharmacists' interventions. Hopefully the continuation of research in this area will further establish clinical settings where pharmacists are needed.

## 4. Conclusion

Currently there are many effective medications used to treat HIV, however these medications may result in significant harm (increased toxicity) or viral resistance when they are not prescribed, administered, or taken correctly [20, 33, 34]. Pharmacists are taking a larger responsibility in caring for patients with HIV, and studies revealing our efforts are increasing. Nonetheless, many of these efforts go undocumented. There are clinical trials published supporting the use of pharmacists in both inpatient and outpatient clinical settings; however, knowledge of the usefulness of these services is often overlooked. Pharmacists are knowledgeable about medications and their management, providing a unique advantage when caring for patients with this disease state. Most studies published showing the effectiveness of pharmacists in treating patients with HIV are limited by their small study size and short duration. Some studies reported outcomes for patients with improvements in CD4+ cells and viral suppression, which can be attributed to the influence of pharmacist recommendations and collaborative efforts. These studies should spur efforts to increase research in using pharmacists as accessible and valuable resources for helping to manage patients with HIV. Another possible area of research could include using pharmacists as resources to enhance continuity of care from the hospital setting to the outpatient clinic setting, acting as guides to provide complete medication reconciliation and possibly decrease drug-related errors. There is also a lack of data researching pharmacy services in areas such as hospice and dialysis centers. These areas are also new realms for research regarding the influence of pharmacists in practice. Pharmacy as a profession is continuously evolving and pharmacists are increasingly seeking opportunities to become more involved in direct patient care. Though there are still many opportunities for research into pharmacists' involvement in care of patients with HIV, pharmacists are currently making vast improvements in the level of patient-centered care they provide for patients living with this complex disease state.

## Figures and Tables

**Table 1:** Pharmacist impact on adherence in patients with HIV/AIDs.

Authors	Year	Analysis type	Objective	No. of patients	Results	Statistical value
Cantwell-McNelis and James [2]	2002	Retrospective	Evaluation of a pharmacist run adherence program	80	(i) Increase in refill rates by patients in contact with a pharmacist (31 versus 50 days)	*P* < 0.05
					(ii) Significant decrease in viral load (values not reported)	*P* < 0.05

Foisy and Akai [3]	2004	Observational, prospective	Describe the implementation of a pharmacy driven direct-observation therapy service	57	(i) 149 drug-related problems identified with 95% acceptance of recommendations (ii) 13.4% drug-related problems included adherence	

Castillo et al. [4]	2004	Retrospective, observational	Compare the impact of different levels of pharmacy care on adherence and time to viral suppression	489	(i) AIDS-tertiary pharmacies had highest rates of adherence compared to outside pharmacies and physician clinics	*P* = 0.000
					(ii) Probability of HIV-1 RNA suppression by 12 months was 74.6% for the AIDS tertiary pharmacies, 59.4% for off site pharmacies, and 60% for physician offices	*P* = 0.001

Hirsch et al. [5]	2009	Cohort	Investigate the impact of pharmacy established MTM services	7,018	(i) 56.3% adherence in pilot pharmacy compared to 38.1% in comparison group	*P* < 0.001
					(ii) Difference in excess refills (19.7% versus 44.8%, pilot pharmacy versus other pharmacies)	*P* < 0.001

Ma et al. [6]	2010	Retrospective, cohort	Investigate clinical outcomes of an HIV clinical pharmacist interventions	75	(i) Prescribed daily pill quantities reduced from a mean of 7.2 ± 3.9 to 5.4 ± 2.8 pills per day	*P* < 0.001
					(ii) 25% increase in CD4+ cell count	*P* < 0.001
					(iii) 33% increase in patients with undetectable viral load	*P* < 0.0001

Henderson et al. [7]	2011	Prospective, cohort	Evaluating antiretroviral adherence and impact of pharmacy interventions	28	(i) Overall 19% increase in adherence rates	*P* < 0.00001
					(ii) Increase in the trend toward undetectable viral load (58–73%, baseline and postintervention)	*P* = 1.0

Hirsch et al. [8]	2011	Cohort	Evaluation of pharmacy driven MTM services	2,234	Increased adherence in the pilot pharmacy than nonpilot pharmacy by 22.1%	*P* < 0.001

MTM: medication therapy management, AIDS: acquired immunodeficiency syndrome, HIV: human immunodeficiency virus.
